# A New Primer Set to Amplify the Mitochondrial Cytochrome *C* Oxidase Subunit I (COI) Gene in the DHA-Rich Microalgae, the Genus *Aurantiochytrium*

**DOI:** 10.1264/jsme2.ME17145

**Published:** 2018-06-01

**Authors:** Goh Nishitani, Masaki Yoshida

**Affiliations:** 1 Graduate School of Agricultural Science, Tohoku University Aoba 468–1, Aramaki, Aoba-ku, Sendai 980–0845 Japan; 2 Faculty of Life and Environmental Sciences, University of Tsukuba 1–1–1 Tennodai, Tsukuba, Ibaraki 305–8572 Japan

**Keywords:** *Aurantiochytrium*, mitochondrial primer, COI

## Abstract

This study was performed in order to develop a primer set for mitochondrial cytochrome *c* oxidase subunit I (COI) in the DHA-rich microalgae of the genus *Aurantiochytrium*. The performance of the primer set was tested using 12 *Aurantiochytrium* strains and other thraustochytrid species. There were no genetic polymorphisms in the mitochondrial sequences from the *Aurantiochytrium* strains, in contrast to the nuclear 18S rRNA gene sequence. This newly developed primer set amplified sequences from *Aurantiochytrium* and closely related genera, and may be useful for species identification and clarifying the genetic diversity of *Aurantiochytrium* in the field.

The marine thraustochytrid genus, *Aurantiochytrium*, produces high levels of polyunsaturated fatty acids (PUFAs), particularly docosahexaenoic acid (DHA). Some *Aurantiochytrium* species are utilized for commercial DHA production, and may also be important components of microbial food webs in marine ecosystems (1, 8). Although species identification is of fundamental importance for research, difficulties are associated with the identification of *Aurantiochytrium* species. Species belonging to this genus have mainly been identified by molecular analyses (the nuclear 18S rRNA gene) due to limited interspecific morphological features. However, several different 18S rRNA gene sequences have been detected despite unialgal clonal cultures in some thraustochytrid strains (6). Many previous studies have used gene cloning in *Escherichia coli* to elucidate *Aurantiochytrium* sequences. However, gene cloning is labor-intensive and costly, and the occurrence of multiple sequences from one strain confuses species identification. In addition, the existence of multiple sequences may result in an overestimation of the genetic diversity of *Aurantiochytrium*. However, in contrast to the nuclear 18S rRNA gene, our preliminary experiments indicated that *Aurantiochytrium* did not show genetic polymorphisms in the sequence of mitochondrial cytochrome *c* oxidase subunit I (COI). The mitochondrial sequences of only two thraustochytrid species are currently registered in GenBank: *Schizochytrium* sp. (accession number KU183024) and *Thraustochytrium aureum* (AF288091 and FJ030898). The sequences of KU183024 (31494 bp) and AF288091 (31570 bp) were obtained as complete or partial mitochondrial genomes, while FJ030898 (684 bp) was obtained by a polymerase chain reaction (PCR) with a mitochondrial COI universal primer set targeting metazoan invertebrates (2). However, the mitochondrial universal primer sequence does not completely match that of *Aurantiochytrium* species, and a suitable PCR amplicon was not obtained in our preliminary experiment. Therefore, a new primer set was developed in the present study in order to sufficiently amplify the mitochondrial COI gene from *Aurantiochytrium*, and the performance of the primer set was examined using cultured strains of *Aurantiochytrium* and other thraustochytrid species.

A new primer set for the genus *Aurantiochytrium* was designed based on the universal primer set (2) and three registered thraustochytrid sequences (KU183024, AF288091, and FJ030898), which are available in GenBank. In order to test the performance of the primer set, 10 unialgal clonal strains (16IR-02, 03, 04, 06, 07, 19, 23, 26, 31, and 43) of *Aurantiochytrium* were isolated according to the method of Wilkens and Maas (10) from a leaf drifting on the seawater surface in a mangrove area, Iriomote Island, Okinawa Prefecture, Japan, in June 2016. We also used an *Aurantiochytrium mangrovei* NYH1 strain previously isolated from Iriomote Island in February 2007, the species identification of which has already been confirmed by an 18S rRNA gene analysis. In order to examine the applicability of the primer set developed in the present study, the following thraustochytrid strains were obtained from the NITE Biological Resource Center (NBRC) and American Type Culture Collection (ATCC): *Aplanochytrium* sp. SEK603 (NBRC 110844), *A. limacinum* SR21 (ATCC MYA-1381), *Botryochytrium radiatum* SEK353 (NBRC 104107), *Labyrinthula* sp. AN-1565 (NBRC 33215), *Oblongichytrium* sp. SEK347 (NBRC 102618), *Parietichytrium sarkarianum* SEK351 (NBRC 104108), *Schizochytrium* sp. SEK210 (NBRC 102615), *Sicyoidochytrium minutum* MBIC11071 (NBRC 102975), *Thraustochytrium* aff. *aureum* SEK621 (NBRC 110820), and *Ulkenia* sp. SEK688 (NBRC 110831).

DNA extraction was performed based on the methods described by Richlen and Barber (9). In order to confirm the existence of multiple sequences, the universal primer set (18S-F1289: TGGAGTGATTTGTCTGGTTRATTCCG, 18SR1772: TCACCTACGGAAACCTTGTTACG) was initially applied to amplify the nuclear 18S rRNA gene (7). PCR conditions are as outlined below. The newly designed primer set, COI-Aur43F (TCTACTAATCAYAAAGATATTGGTACT) and COI-Aur748R (TCAGGATGACCAAAAAACCA), was used to amplify the mitochondrial COI gene. This primer set generated a single band of 705 bp from *Aurantiochytrium* DNA. PCR was performed using a Veriti thermal cycler (Thermo Fisher Scientific, Waltham, MA, USA) in a 20-μL reaction mixture containing 1.0 μL of template DNA, 0.2 mM of each dNTP, 1×PCR buffer, 1.5 mM Mg^2+^, 1.0 U KOD-Plusver. 2 (Toyobo, Osaka, Japan) with intensive 3′→5′ exonuclease activity, and 0.2 μM of each primer. The PCR cycling conditions employed were as follows: initial denaturation at 94°C for 2 min followed by 32 cycles of 98°C for 10 s, 54°C for 30 s, and 68°C for 45 s. PCR products were then purified using ExoSAP-IT PCR product cleanup reagent (Thermo Fisher Scientific). DNA sequences were examined directly without gene cloning using a DYEnamic ET Terminator Cycle Sequencing Kit (GE Healthcare, Little Chalfont, Buckinghamshire, UK) and analyzed on a 3730xl DNA Analyzer (Thermo Fisher Scientific). The forward and reverse sequences were aligned using GENETYX software (Genetyx Corporation, Tokyo, Japan). Partial sequences of the mitochondrial COI gene were aligned and an unrooted phylogenetic tree was generated with the maximum likelihood (ML) method using MEGA version 7 software (4) with the default settings. The topology of the phylogenetic tree was evaluated by the bootstrap method with 100 replicates.

In the 18S rRNA gene analysis using the universal primer, the sequences of 10 newly isolated *Aurantiochytrium* strains, *A. mangrovei* NYH1, *A. limacinum* SR21 (ATCC, MYA-1381), and *Schizochytrium* sp. SEK210 (NBRC, 102615) were not elucidated by direct sequencing. This may have been due to the influence of multiple sequences of the 18S rRNA gene in thraustochytrids, as has been suggested by Nakazawa *et al.* (6).

PCR amplification using the mitochondrial COI gene primer set designed in the present study produced a clear single band from the following DNA sources: 10 *Aurantiochytrium* strains, *A. mangrovei* NYH1, *A. limacinum* SR21, *B. radiatum*, *P. sarkarianum*, *Schizochytrium* sp., and *Thraustochytrium* aff. *aureum* (Fig. 1). These mitochondrial sequences were elucidated without gene cloning, suggesting that the mitochondrial region is suitable for species identification and a genetic diversity analysis of the genus *Aurantiochytrium* and closely related genera.

A phylogenetic tree of the mitochondrial COI gene was generated based on the sequences of 16 thraustochytrid strains obtained in the present study and available sequence data from GenBank, with two *Laminariocolax* species included as an outgroup (Fig. 2). All *Aurantiochytrium* strains were grouped into four clusters based on the phylogenetic tree: (i) *A. limacinum*, (ii) *A. mangrovei*, (iii) *Aurantiochytrium* sp. (strain 16IR-43), and (iv) *Aurantiochytrium* sp. (strain 16IR-02, 03). The *A. limacinum* cluster contained six Japanese strains (16IR-06, 07, 19, 23, 26, and 31), *A. limacinum* SR21 from Micronesia (3, 5, 11), and *Schizochytrium* sp. from China. The sequences of the Japanese and SR21 strains had a difference of 2/660 bases, which may represent an intraspecific difference. The sequence of the 16IR-04 strain was identical to that of *A. mangrovei*. Another three strains (16IR-02, 03, and 43) were confirmed to be identical to *Aurantiochytrium* sp. (AB811029) by a partial 18S rRNA gene analysis (data not shown).

In conclusion, the mitochondrial primer set for *Aurantiochytrium* designed in the present study has two benefits: (i) it does not generate multiple sequences from a single clonal strain, suggesting that direct sequencing is possible; and (ii) it amplifies the DNA of the genus *Aurantiochytrium* and closely related genera, suggesting that these primers are applicable to not only culture strains, but also field samples in order to elucidate the distribution and true diversity of *Aurantiochytrium*.

The mitochondrial COI gene sequences obtained in the present study have been deposited in the DDBJ/EMBL/GenBank databases under accession numbers LC318552–LC318560 and LC333576.

## Figures and Tables

**Fig. 1:**
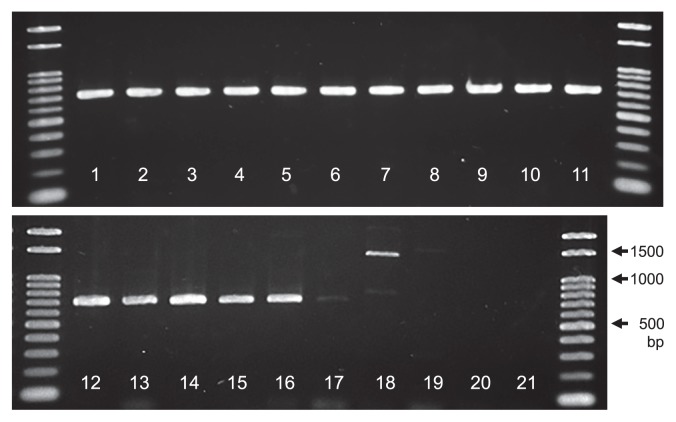
PCR products of thraustochytrids obtained using the mitochondrial COI primer set (COI-Aur43F/COI-Aur748R). The strains tested were: Lanes 1–11, *Aurantiochytrium* 16IR-02, 03, 04, 06, 07, 19, 23, 26, 31, 43, and *A. mangrovei* NYH1, respectively; lane 12, *A. limacinum* SR21; lane 13, *Schizochytrium* sp. SEK210; lane 14, *Thraustochytrium* aff. *aureum* SEK621; lane 15, *Botryochytrium radiatum* SEK353; lane 16, *Parietichytrium sarkarianum* SEK351; lane 17, *Oblongichytrium* sp. SEK347; lane 18, *Labyrinthula* sp. AN-1565; lane 19, *Sicyoidochytrium minutum* MBIC11071; lane 20, *Ulkenia* sp. SEK688; lane 21, *Aplanochytrium* sp. SEK603.

**Fig. 2:**
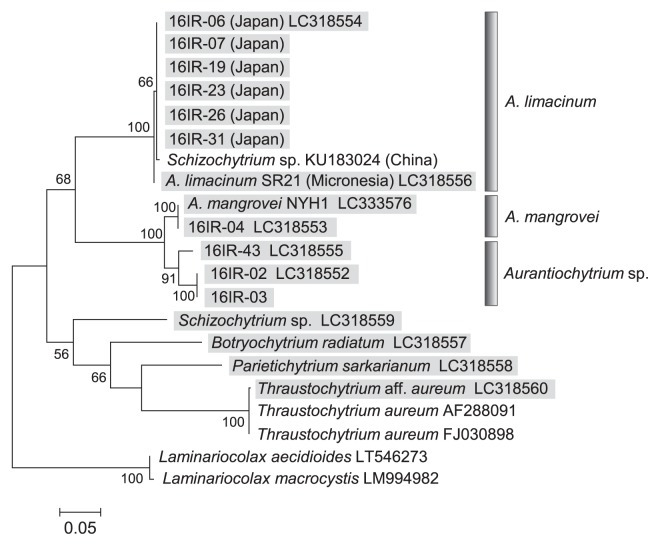
Maximum likelihood (ML) tree for the mitochondrial COI gene of thraustochytrids analyzed in the present study along with other thraustochytrid sequences from GenBank using MEGA software ver. 7. Sequences elucidated in the present study are indicated by gray highlighting with accession numbers. Bootstrap values of >50% are given as percentages of 100 bootstrap replicates at the respective nodes. The final dataset contained 657 informative sites. The scale bar represents the number of substitutions per site.
